# Low concentrations of acetic and formic acids enhance the inactivation of *Staphylococcus aureus* and *Pseudomonas aeruginosa* with pulsed electric fields

**DOI:** 10.1186/s12866-019-1447-1

**Published:** 2019-04-03

**Authors:** Vitalij Novickij, Eglė Lastauskienė, Gediminas Staigvila, Irutė Girkontaitė, Auksė Zinkevičienė, Jurgita Švedienė, Algimantas Paškevičius, Svetlana Markovskaja, Jurij Novickij

**Affiliations:** 1 Faculty of Electronics, Vilnius Gediminas Technical University, Naugarduko st. 41, 03227 Vilnius, Lithuania; 2 Institute of Biosciences, Life Sciences Centre, Vilnius University, Sauletekio al. 7, 10257 Vilnius, Lithuania; 3 Department of Immunology, State Research Institute Centre for Innovative Medicine, Santariškių st. 5, 08406 Vilnius, Lithuania; 4 Laboratory of Biodeterioration Research, Nature Research Centre, Akademijos st. 2, 08412 Vilnius, Lithuania; 5 Laboratory of Mycology, Nature Research Centre, Žaliųjų ežerų st. 49, 08406 Vilnius, Lithuania

**Keywords:** Bacteria, Wound healing, Electroporation, Infection control, Electropermeabilization

## Abstract

**Background:**

Skin infections, particularly caused by drug-resistant pathogens, represent a clinical challenge due to being a frequent cause of morbidity and mortality. The objectives of this study were to examine if low concentrations of acetic and formic acids can increase sensitivity of *Staphylococcus aureus* and *Pseudomonas aeruginosa* to pulsed electric field (PEF) and thus, promote a fast and efficient treatment methodology for wound treatment.

**Results:**

We have shown that the combination of PEF (10–30 kV/cm) with organic acids (0.1% formic and acetic acids) increased the bactericidal properties of treatment. The effect was apparent for both acids. The proposed methodology allowed to reduce the energy of electrical pulses and the inhibitory concentrations of acids, while still maintain high efficiency of bacteria eradication.

**Conclusions:**

Application of weak organic acids as bactericidal agents has many advantages over antibiotics because they do not trigger development of drug-resistance in bacteria. The combination with PEF can make the treatment effective even against biofilms. The results of this study are particularly useful for the development of new methodologies for the treatment of extreme cases of wound infections when the chemical treatment is no longer effective or hinders wound healing.

## Background

Skin infections, particularly caused by drug-resistant pathogens, represent a clinical challenge due to being a frequent cause of morbidity and mortality [1, 2]. The complications from the non-healing wounds may include septicemia, long-time hospitalization, chronic pain or limb amputations [3–6]. Burn wound infections are associated with even more severe complications and high mortality rates [7]. Multiple bacterial species can be responsible for burn wound infections, with the *Staphylococcus aureus* and *Pseudomonas aeruginosa*, being the most common [4, 8].

*S. aureus* is a major human pathogen, which will likely remain both common and serious due to the high environmental adaptability and development of resistance to antibiotics [9–11]. Infections due to *S. aureus* isolates with resistance to vancomycin have been associated with multiple treatment failures [11, 12]. At the same time, *P. aeruginosa* can be associated with refractory skin ulcer, bacterial biofilm formation and resistivity to the majority of antimicrobials [13, 14]. Since the biofilms are resistant to antibiotics and host defenses, targeting the pathogen with other antibacterial methods may be the only option to promote wound healing [4, 15]. For example, application of antimicrobial peptides or synthesized nanoparticles could be considered [3, 16, 17]. However, most of the studies have focused on the research of chemical bactericidal agents, which are weak against biofilms, therefore physical methods started to emerge [13, 18, 19]. One of the methodologies, which has shown a high potential for wound sterilization and healing is electroporation [20–22]. The electroporation is a pulsed electric field (PEF) induced phenomenon, which triggers permanent or reversible permeability of cells to other molecules [23, 24]. The first studies, which highlighted the potential of electroporation for treatment of surface infections were published within the past 5 years, showing positive results for eradication of the skin infections causative microorganisms [20, 22, 25, 26]. Currently, most of the studies on bacteria and yeast treatment with PEF are focused on protein extraction, food processing and transformation methods [27–29], while biomedical application are dominated by cancer treatment [30–33]. Therefore, application of PEF for wound sterilization is an emerging field, while PEF-based wound healing techniques have been proposed earlier [34, 35]. The main reason for the delay in the area of PEF wound sterilization could be the dependence of electroporation efficiency on the pulse parameters [36, 37]. Bacteria and yeast are way more resistant to PEF if compared to mammalian cells, therefore higher requirements for development of pulse generators to trigger the electroporation phenomenon in prokaryotes is required [38–41]. However, with the development of the pulsed power technologies and increased availability of the electroporators, the first studies on the interactions of the bacterial cell wall and PEF started to appear [42]. Also, it was shown that the permeabilization of the bacteria membranes allowed restoring the sensitivity of pathogens to antimicrobial agents, which is of high relevance in the area of wound healing and treatment of the surface infections [16].

In this study, we focused on the inactivation efficiency of electroporation in a broad range of PEF amplitudes (10–30 kVcm^− 1^) for both the gram-positive *S. aureus* and gram-negative *P. aeruginosa*. Taking into account the recent advances in the understanding of bacteria cell wall interactions with PEF [42] and the varied susceptibility of bacteria to antimicrobials due to increased permeabilization [16], we have combined electroporation with weak organic acids (i.e. acetic and formic) in order to induce better inactivation. Acetic acid in low concentrations has already proven to be a potent antiseptic, especially when common antiseptics (hydrogen peroxide, iodine and alcohol) are toxic to the cells, which are involved in wound healing [43–45]. We presumed, that when combined with electroporation the methodology is very potent for treatment of burn wound infections, where both high inactivation efficiency and wound healing is required. The low concentration formic acid has been used as a reference due to its low toxicity [46]. Since electroporation is highly associated with reactive oxygen species and oxidation mechanisms [47–49], we wanted to test if the additive effect of acetic acid with PEF is apparent with other acids. The PEF induced cell permeabilization experiments have been performed in vitro and the inactivation efficiency was evaluated.

## Methods

### Cultivation of bacterial cells and determination of minimal inhibitory concentrations of acids

*S. aureus* (ATCC29213) and *P. aeruginosa* (ATCC27853) cells were grown over night in liquid LB (Luria-Bertani) medium (10 g/l tryptone, 5 g/l yeast extract, 5 g/l NaCl) in the rotary shaker at 37 °C. 1 ml of the OD = 1 (600 nm; 1.5 × 10^9^ cells) cell culture was transferred to 10 ml of the fresh LB medium and grown 4 h at the same conditions. Later the cells were washed 3 times with 1 M sorbitol and re-suspended in 1 M sorbitol at final concentration of 10^9^.

The minimal inhibitory concentrations (MIC) of acetic and formic acid were determined by using agar diffusion and microdilution methods in 96-well microdilution plates. For agar diffusion, *S. aureus* and *P. aeruginosa* cells were subcultured on the LB agar medium at 37 °C. Cell suspensions were prepared using liquid LB medium (OD = 1). Inocula were spread on solid agar and 3 mm holes were punched in the agar plates. 1, 0.5, 0.3, 0.2 and 0.1% of formic and acetic acids were added to the wells. Plates were analyzed individually by measuring the inhibition zone.

For the microdilution method, the cell suspension was prepared as described above and 100 μl of the cell suspension was added to the 100 μl of LB medium supplemented with the known concentrations of tested compounds. The 100 μl of each bacteria suspension was added to the 100 μl LB medium as a positive control. Medium alone was used as a sterile control. Microplates were incubated at 37 °C for 24 h. The first 2 wells without visible growth were counted as MIC and plated on the LB agar plates at 37 °C for 24 h as a reference.

All experiments were performed in triplicate.

### Electroporation

The square wave electroporator capable of generating 100 ns – 1 ms pulses up to 3 kV was used in the study [50]. The commercial electroporation cuvette (VWR International, Radnor, PA, USA) with 1 mm gap between the electrodes was used as a load. For bacteria inactivation, the pulses were generated in bursts of 10 or 20 with a fixed duration of 100 μs, while for the permeabilization experiments, single 100 μs pulses were used. The PEF amplitude was varied in the range of 10–30 kVcm^− 1^, corresponding to 1–3 kV voltage drop on the electroporation cuvette. Samples of 70 μL of the cell suspension (prepared in 1 M sorbitol) were used for electroporation.

For combined PEF and chemical treatment, the 63 μL of cell suspension was mixed with 7 μL of 1% acid (either acetic or formic) resulting in a final 0.1% concentration. The resulting suspension was transferred to cuvette and the 10 or 20 pulses protocols were applied, followed by a 3-min incubation in room temperature.

### Flow cytometry

The samples of the cell suspension (63 μL) were mixed with 7 μL propidium iodide (PI) (Sigma-Aldrich, Germany) at final concentration of 50 μM [51] and transferred to electroporation cuvette for pulse application. After the pulsing, a 10-min incubation at room temperature was performed for passive dye diffusion, followed by flow cytometric analysis. The samples were analyzed by FlowSight (Amnis, Seattle, USA) flow cytometer. The fluorescent cells (PI permeable) were gated as permeabilized in accordance with standard gating strategy used in electroporation studies [51–53].

### Cell survival

After electroporation, the *S. aureus* and *P. aeruginosa* cells were plated on LB agar. After 24 h of growth at 37 °C the colony forming units (CFU) were counted. As a reference, the untreated control samples were used (CFU_C_). The inactivation efficiency was evaluated as a ratio between the treated sample (CFU_T_) and the control (CFU_C_) expressed as a percentage.

For the combined PEF and chemical treatment, firstly the MICs of acids were determined. Agar diffusion and microdilution showed a 0.2% MIC using both acids for both bacteria species. Therefore, we have used a smaller concentration of 0.1% (both for acetic and formic acid) to minimize the effect of timing during experiment. As a result, the induced inactivation effects purely due to chemical treatment were also minimized. After pulse application, the cells were incubated in room temperature for 3 min and plated on LB agar plates.

### Statistical analysis

One-way analysis of variance (ANOVA; *P* < 0.05) was used to compare the differences in efficiency between protocols. If ANOVA indicated a statistically significant result, Tukey HSD multiple comparison test was used (*P* < 0.05 was considered statistically significant). The data was post-processed using OriginPro 8.5 software (OriginLab, Northhampton, MA, USA). All experiments have been performed at least in triplicate and all values are presented as mean ± standard deviation.

## Results

### Permeabilization of bacteria in pulsed electric field

**Fig. 1:**
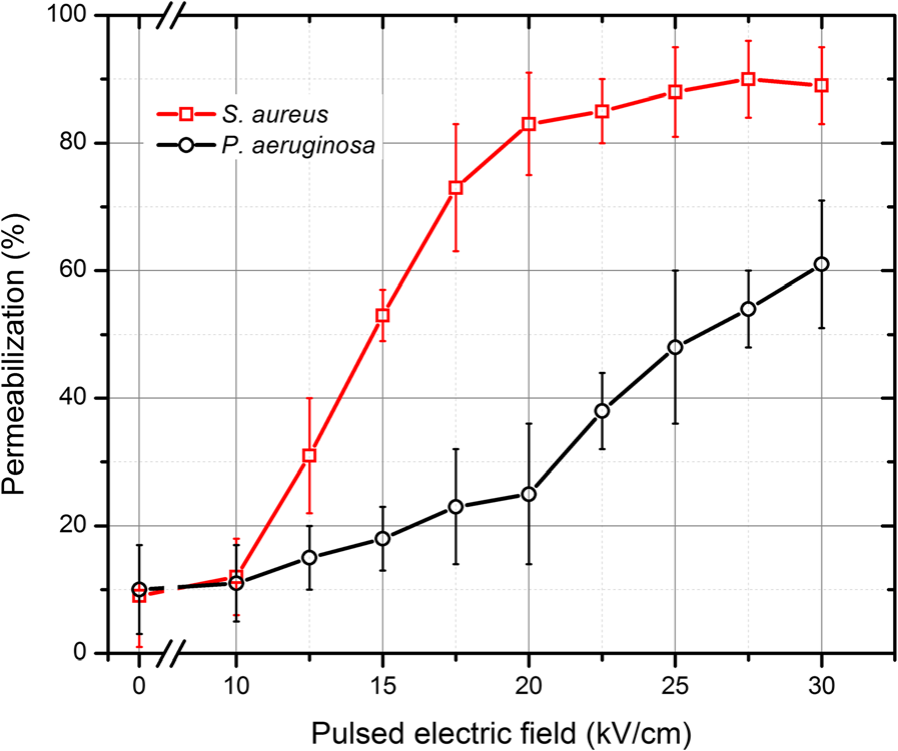
The permeabilization of *S. aureus* and *P. aeruginosa* during pulsed electric field treatment. The *S. aureus* was more susceptible and the 20 kVcm^− 1^ × 100 μs protocol induced a saturated (> 85%) effect

The permeabilization results of *S. aureus* and *P. aeruginosa* are presented in Fig. 1. As expected, the gram-negative *P. aeruginosa* was less susceptible to PEF if compared with the gram-positive *S. aureus*. The weaker effect of PEF on *P. aueruginosa* was apparent (*P* < 0.05) for all amplitudes in the 12.5–30 kVcm^− 1^ range. Even after 30 kVcm^− 1^ single 100 μs pulse less than 70% cells were permeable to PI, while the *S. aureus* reached a saturated permeabilization (> 85%) already after the 20 kVcm^− 1^ PEF treatment. The number of permeabilized cells in the control samples without any treatment did not exceed 10 ± 8% for both bacteria.

### Survival of bacteria after electroporation

**Fig. 2:**
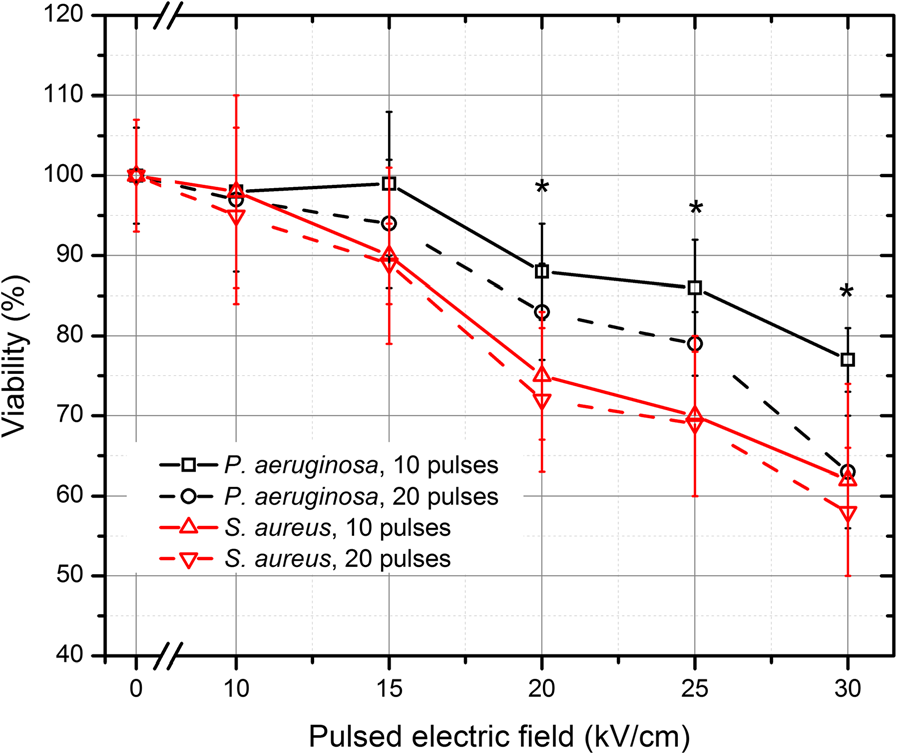
The dependence of *S. aureus* and *P. aeruginosa* viability on the applied pulsed electric field parameters. Higher number of pulses resulted in higher loss of viability for both bacteria, however the effect was more profound (*P* < 0.05) in the *P. aeruginosa* case. The asterisk (*) represents statistically significant difference (*P* < 0.05) versus control (non-treated samples) for both bacteria

The viability data on bacteria after PEF treatment are summarized in Fig. 2. It should be noted, that the permeabilization induced by single 100 μs pulse was reversible (effect on cell survival was negligible), therefore data for 10 and 20 pulses bursts are presented. As it can be seen in Fig. 2, the same tendency of *S. aureus* being more susceptible to treatment persists, however the difference in viability from *P. aeruginosa* was statistically significant only in the 20–30 kVcm^− 1^ range. More than 40% inactivation rate was achieved (*S. aureus*, 30 kVcm^− 1^). We could have increased the total number of applied pulses and thus induce higher inactivation, however the goal was to detect additive effects with chemical agents. The reduction of viability in the 25–40% range was considered optimal and guaranteed high permeabilization of bacteria (see Fig. 1). We have applied the same inactivation protocols (10 and 20 pulses) in combination with low concentration of acids.

### Survival of bacteria after combined treatment with acids

**Fig. 3:**
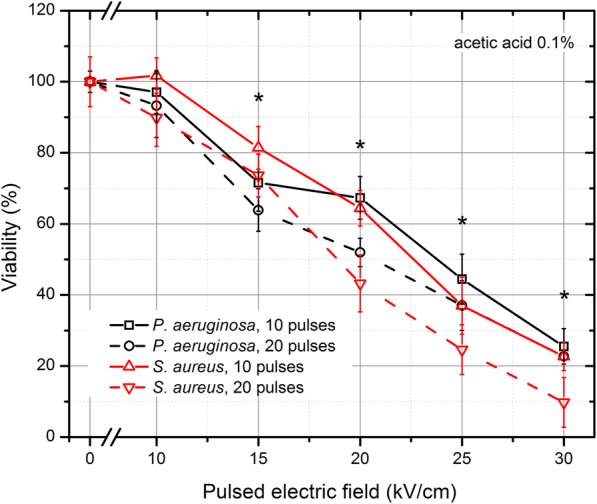
The inactivation of bacteria using pulsed electric field treatment combined with acetic acid (0.1%). The treatment was efficient for both bacteria, which was unachievable during separate exposures. The asterisk (*) represents statistically significant difference (*P* < 0.05) versus control (chemical treatment only)

**Fig. 4:**
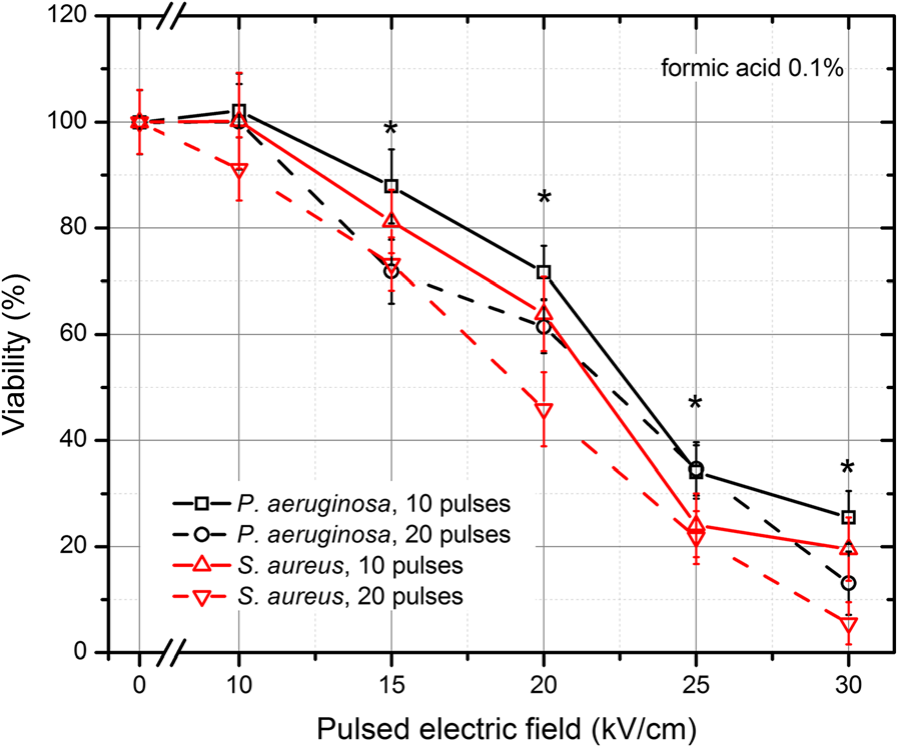
The inactivation of bacteria using pulsed electric field treatment combined with acetic acid (0.1%). The treatment was efficient for both bacteria, which was unachievable during separate exposures. The asterisk (*) represents statistically significant difference (*P* < 0.05) versus control (chemical treatment only)

The results for acetic acid (0.1%) are presented in Fig. 3. As it can be seen in Fig. 3 the inactivation rate was significantly higher for both bacteria when the PEF method was applied in combination with low concentration (0.1%) of acetic acid. The same methodology was applied with PEF and formic acid (0.1%). The results are summarized in Fig. 4. It can be seen that a similar tendency of inactivation was also apparent. The *S. aureus* was more susceptible to the treatment, while the survival of bacteria was not affected (*P* > 0.05) by the 10 kVcm^− 1^ protocol.

**Fig. 5:**
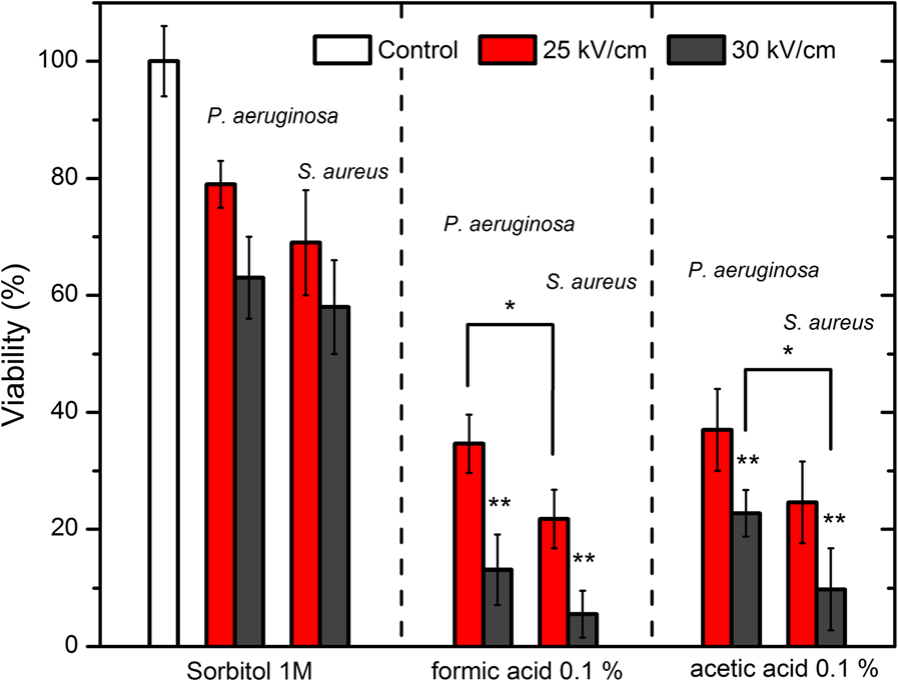
The comparison of bacteria inactivation induced by the most effective protocols that were proposed in the study. The asterisk (*) represents statistically significant difference (*P* < 0.05) between *S. aureus* and *P. aeruginosa*. The asterisk (**) represents statistically significant difference (*P* < 0.05) between 25 and 30 kVcm^− 1^ procedures, which indicates that the effect is not saturated and can be enhanced

The comparison of the most effective PEF protocols (20 pulses; 25–30 kVcm^− 1^) using both acids is presented in Fig. 5. The combination of chemical treatment with PEF resulted in a significant improvement of treatment efficiency for both bacteria. Also, there was statistically significant difference between *P. aeruginosa* and *S. aureus* susceptibility to the combined treatment for both acids (*). Formic acid on average was more potent than acetic acid resulting in almost full eradication of *S. aureus* when combined with PEF. The increase of bacteria membrane permeabilization by PEF induced sensitivity to the both acids, which was not detectable when acids were used in low concentrations.

## Discussion

Application of weak organic acids as bactericidal agents has many advantages over antibiotics because they do not trigger development of drug-resistance in bacteria [43]. Concentrations > 2% have been frequently used to overcome infection in infected burn or chronic wounds both in animal and human models [45, 54]. Also in 2016, Golberg et al. successfully used electroporation to eradicate pathogenic bacteria in wounds [20] and it was also shown that it does not impair wound healing [35]. In our work, we have combined the two potent methodologies (electroporation and acids) and have shown that it is possible to further increase the sensitivity of bacteria to chemical agents.

The possible mechanism of the effect (bacteria sensitization) presumably lies within the drastic physical damage that is caused by PEF. The exposure to PEF triggers permeabilization of plasma membrane and disruption of the cell wall integrity [27, 42]. The cell wall is the main bacteria’s barrier against environment and the cell wall-targeting antibiotics are a common solution to overcome the drug-resistivity when used in combination therapies [55, 56]. However, in the electroporation case it is a physical method, which is non-toxic and can be successfully used even for eradication of biofilms [22]. The combination of PEF with organic acids increased the bactericidal properties of treatment, which can be attributed to the internal pH shock, which was induced due to the high permeabilization of bacteria cell wall and uptake of acids [45, 54]. The effect was apparent for both acids (acetic and formic), which supports the hypothesis and is in agreement with previously observed effects in yeast [57]. Also, the oxidative stress and reactive oxygen species (ROS) may have significantly contributed to the lethality of the treatment [58, 59]. The proof of concept that PEF can generate both extracellular (electrochemical) and intracellular ROS was reported before [49]. The increase of intracellular O_2_ can cause oxidative stress in bacteria, which is extremely harmful to microorganisms and a number of enzymes with active-site iron-sulfur clusters are highly sensitive to inactivation by O_2_ [59].

Nevertheless, electroporation is a pulse dependent phenomenon and the inactivation of bacteria solely by PEF may result in thermal damage of tissue, pain or muscle contractions [60, 61], while high concentration of acid (> 2%) may cause significant pain [45, 54]. In this work, we have used acid concentrations, which are below the minimal inhibitory dose, however the additive effect with PEF was still detectable. The result is of high relevancy since the bacteria sensitization phenomenon allows to reduce both the energy of electrical pulses and the concentration of acids, while still maintain high efficiency of bacteria eradication. Similar to Golberg et al., we have used microsecond pulses, however we have applied shorter bursts (lower number of pulses) for a faster procedure. It should be noted that microsecond range protocols (10–30 kV/cm) may result in high muscle contractions in vivo and due to different susceptibility of mammalian and bacterial cells to PEF, healthy tissue can be also affected [20]. Therefore, adopting of the proposed methodology in the nanosecond PEF range is more advantageous from the clinical perspective. The nanosecond range PEF protocols will allow reduction of the muscle contractions [62], ensure a more homogenous exposure [63] and better energy control [64]. The optimization of the protocols should be performed.

Firstly, the amplitude and/or duration of PEF pulses should be reduced to prevent/minimize damage of healthy tissue and muscle contractions, which was also highlighted by Golberg et al [20]. Further, the pulse delivery methodology can be altered. We believe, that the potential solution to the mentioned problems is to use the topical delivery of acetic acid to serve as a high impedance electrode/tissue interface and deliver the pulses using electrodes without forming a skin lump or keeping it minimal. The difference in load bioimpedances would result in only a thin layer of epidermis being affected, while the depth of ablation could be controlled by the bioimpedance of the electrode/tissue contact and by the amplitude of electric field [65]. In cases of wound treatment, the low penetration depth of PEF is advantageous, since it will allow to sustain the PEF effect predominantly in the infected volume. The problem and the requirement for penetration of deep tissues are specific for cancer treatment applications [66].

## Conclusions

It was shown that electroporation in combination with acetic and formic acid triggers inactivation of *S. aureus* and *P. aeruginosa.* Taking into account the advantages of organic acids over antibiotics, the proposed methodology has high potential applicability. The results are useful as a starting point and as a proof of concept for the development of new PEF-based methods for the treatment of extreme cases of wound infections, especially when the chemical treatment is no longer effective or hinders wound healing. The future in vivo works should involve optimization of the PEF parameters and determination of the optimal acid concentration to ensure painless, fast and efficient procedure. Lastly, the efficacy of the PEF methodology for the treatment of biofilms should be investigated and is a matter of future works.
